# Inhibition of Pathological Retinal Neovascularization by a Small Peptide Derived from Human Tissue-Type Plasminogen Kringle 2

**DOI:** 10.3389/fphar.2019.01639

**Published:** 2020-01-28

**Authors:** Qian Sun, Yinchen Shen, Li Su, Xun Xu

**Affiliations:** ^1^Department of Ophthalmology, Shanghai General Hospital (Shanghai First People’s Hospital), Shanghai Jiao Tong University School of Medicine, Shanghai, China; ^2^Shanghai Key Laboratory of Fundus Disease, Shanghai Jiao Tong University, Shanghai, China; ^3^Shanghai Engineering Center for Visual Science and Photomedicine, Shanghai, China

**Keywords:** peptides, tissue-type plasminogen activator, kringle, retinal angiogenesis, angiogenesis inhibitor

## Abstract

Retinal neovascularization is a hallmark pathological process of numerous ocular diseases which comprise the most common causes of blindness and affect millions of people from infants to the elderly. Compared to large proteins, small peptides have advantages for therapeutic application in ocular diseases, especially for retinal diseases. In this study, we investigated a small peptide derived from human tissue-type plasminogen kringle 2 (t-PA kringle 2), named TKII-12, and investigated the effect of TKII-12 on various aspects of vascular endothelial growth factor (VEGF)-induced angiogenesis *in vitro* and *in vivo*. Our results showed that TKII-12 effectively inhibited VEGF-induced human retinal microvascular endothelial cell proliferation, migration and tube formation on Matrigel dose-dependently as well as sequence-dependently. TKII-12 inhibited VEGF-induced formation of actin stress fibers and focal adhesions in vascular endothelial cells. Moreover, TKII-12 effectively inhibited retinal neovascularization in a mouse oxygen-induced retinopathy (OIR) model. Our study demonstrated that TKII-12 could effectively inhibit retinal angiogenesis *in vitro* and *in vivo* by eliminating the formation of focal adhesion complexes and the organization of actin stress fibers. TKII-12 can serve as a prototype for retinal angiogenesis inhibitory drug development.

## Introduction

Retinal neovascularization is a hallmark pathological process of numerous ocular diseases, such as proliferative diabetic retinopathy, ischemic retinal vein occlusion, and retinopathy of prematurity. Abnormal retinal neovascularization always leads to hemorrhage, retinal edema, fibrosis, and irreversible damages to the retinal tissue. Neovascular retinopathies collectively comprise the most common causes of blindness and affect millions of people from infants to the elderly (Lad et al., 2009; Wong et al., 2014; Lee et al., 2015; Ho et al., 2016).

In the past two decades, antivascular endothelial growth factor (VEGF) medications for neovascular ocular diseases have revolutionized the treatment paradigm for millions of patients and preserved their vision. However, there remain pitfalls and areas for improvement with these anti-VEGF therapies. Currently, the most widely used retinal angiogenesis inhibitors, such as ranibizumab, aflibercept, and bevacizumab, are large and complex proteins, that are difficult to scavenge and expensive to manufacture (Avery et al., 2017). Compared to these proteins, small peptides have advantages for therapeutic application, due to their high solubility, increased bio-availability and lack of immune response in the host cell. Furthermore, the production of small peptides is controllable and much easier than that of proteins. Thus, designing and developing peptides for therapeutic application to inhibit angiogenesis is an important area in antiangiogenic drug development (Sulochana and Ge, 2007; Kaspar and Reichert, 2013; Agyei et al., 2017; Lau and Dunn, 2018).

Kringle domains are protein modules composed of 78–80 amino acids connected by a characteristic triple disulfide-linked loop. To date, many kringle-containing fragments from various endogenous proteins have been reported to inhibit angiogenesis *in vitro* and *in vivo*, such as angiostatin from plasminogen, KIV-9, KIV-10, and KV from human apolipoprotein (a), kringle 1 from hepatocyte growth factor, kringle-2 from prothrombin, and kringle 1-2 and K2P from human tissue-type plasminogen activator (t-PA) (Lim et al., 2012; Cho et al., 2013; Sun and Liu, 2019). Thus, the kringle structure has been considered as a conserved architecture that specifically inhibits angiogenesis. Kringle domains may provide a structural basis for identification of novel angiogenesis inhibitors.

**Figure 1:**
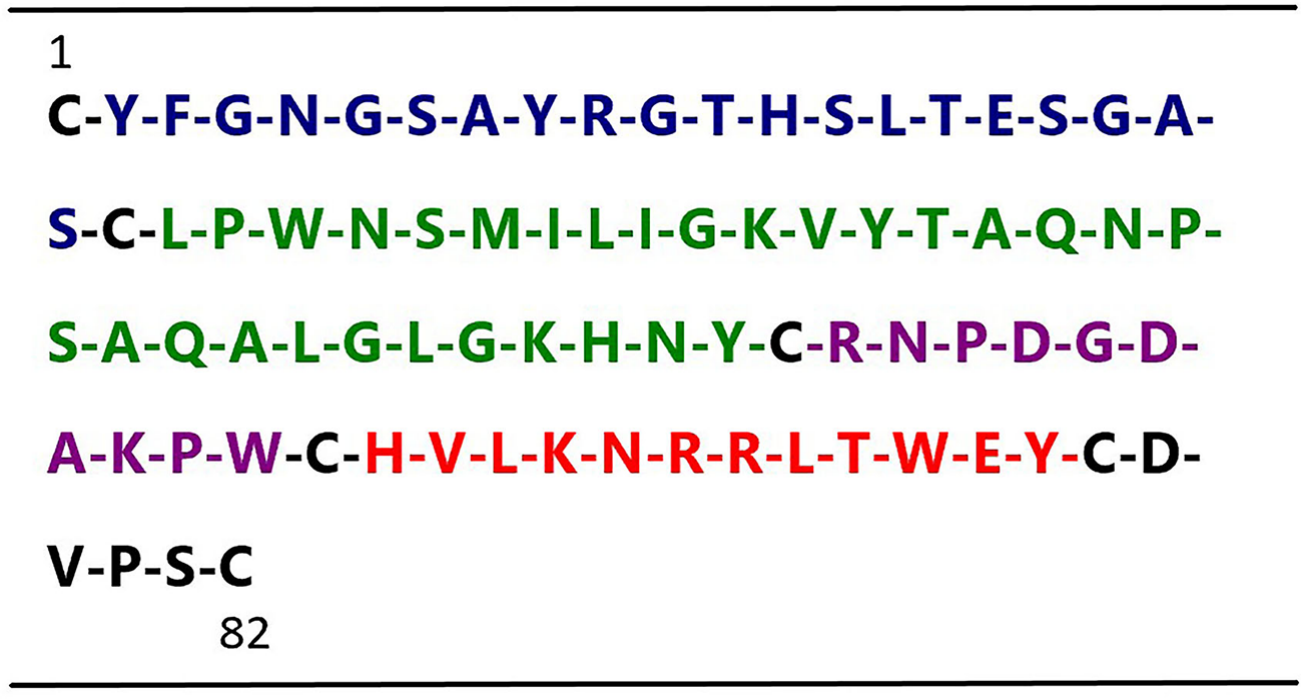
Sequence of human t-PA kringle 2. The figure shows the primary sequences of the four t-PA kringle 2-derived peptides, highlighted in blue Peptide 1 (Tyr2–Ser21), green Peptide 2 (Leu23–Tyr52), purple Peptide 3 (Arg54–Trp63) (named TKII-10), and red Peptide 4 (His65–Tyr76)(named TKII-12), respectively.

In an effort to develop small peptide angiogenic inhibitors, we divided human t-PA kringle 2 amino acid sequences into 4 nonoverlapping small peptides by using the cysteine in disulfide bonds for cleavage sites (not including cysteine) (**Figure 1**). Our previous study demonstrated that one of these four peptides, TKII-10, which consisted of the Arg54-Trp63 amino acid of human t-PA kringle 2, inhibited VEGF stimulated migration and tube formation of human umbilical vein endothelial cells (HUVECs) *in vitro* and effectively inhibited angiogenesis in chick chorioallantoic membrane and VEGF-induced corneal neovascularization (Su et al., 2010).

In this study, we investigated another small peptide derived from human t-PA kringle 2, named TKII-12, and explored the antiangiogenic effects of TKII-12 *in vitro* and *in vivo*, in an effort to search for a more effective antiangiogenic agent for the treatment of neovascular retinopathies. MTS assay, Transwell chamber assay and tube formation assay were performed to determine the inhibitory effects of TKII-12 on VEGF-induced vascular endothelial cells proliferation, migration, and tube formation *in vitro*. The *in vivo* antiangiogenic effect of TKII-12 was evaluated in mice with oxygen-induced retinopathy (OIR). The formation of actin stress fibers and focal adhesions in vascular endothelial cells were investigated to explore the antiangiogenic mechanism of TKII-12.

## Materials and Methods

### Animals

All animals were cared for in accordance with the ARVO Statement for the Use of Animals in Ophthalmic and Vision Research. Neonatal C57BL/6J mice with nursing mothers were provided by Shanghai Laboratory Animal Center, Chinese Academy of Sciences. The animals were housed in an air-conditioned room with a 12-h light and dark cycle.

### Cell Culture and Materials

HRMECs (cat. no. ACBRI 181) were purchased from Cell Systems (Kirkland, WA) and cells from passages 3–7 were used in the experiment. Cells were grown in M199 medium with 45 ng/ml bFGF and heparin and 20% fetal bovine serum. Confluent cells were switched to a serum-free medium for 24 h before treatment. Human VEGF165 was purchased from R&D Systems, Inc. (Minneapolis, MN). The Transwell chamber (8.0-µm pore size) was purchased from Costar (Corning, Cambridge, MA). Growth factor-reduced Matrigel was purchased from BD Biosciences (Bedford, MA).

### Preparation of Peptides

The peptides TKII-12 (HVLKNRRLTWEY) and TKII-10 (RNPDGDAKPW) were synthesized by a high-efficiency solid-phase method using an automatic peptide synthesizer (Symphony; Protein Technologies, Tucson, AZ). The end product was characterized by high-performance liquid chromatography (HPLC, analytical; Shimadzu, Kyoto, Japan) and mass spectrometry (MS; Finnigan TSQ 7000; Thermo, Waltham, MA). To verify whether the effect of TKII-12 was sequence-dependent, we scrambled the amino acid sequence of TKII-12 and synthesized TKII-12S (KRYLTHNVRWLE). These peptides were freeze-dried and stored at −20°C until used. Immediately before use, the peptides were dissolved in phosphate-buffered saline (PBS). Both these peptides were water-soluble and stable in aqueous solutions.

### Endothelial Cell Proliferation Assay

Endothelial cell proliferation assay was determined using the nonradioactive CellTiter 96^®^ aqueous one solution (Promega, Madison, WI). Briefly, approximately 3,500 cells/well were added in triplicate into each well of 96-well cell culture plates and incubated at 37°C for 24 h. After 24 h, HRMECs were starved overnight and then incubated with or without 10 ng/ml of VEGF and various concentrations of peptide (1 nM, 10 nM, 100 nM, 1 µM, and 10 µM) for 24 h. Then, 20 µl of CellTiter 96^®^ AQueous One Solution was added to each well and incubated for another 3 h at 37°C. The absorbance at 490 nm, which correlates to the number of living cells, was measured with a microplate reader (Bio-Rad, Model 680, Hercules, CA). Each group was tested in triplicate. All the experiments were repeated 3 times.

### Endothelial Cell Migration Assay

To determine the effect of TKII-12 peptide on endothelial cell migration toward VEGF, endothelial cell migration assay was performed using a disposable Transwell chamber as described previously with modifications (Sulochana et al., 2005). Briefly, HRMECs were starved overnight, trypsinized, and suspended at a final concentration of 8×10^5^ cells/ml. Various concentrations of peptide (1 nM, 10 nM, 100 nM, 1 µM, and 10 µM) were preincubated with approximately 4×10^4^ cells for 30 min at 37°C before seeding onto the cell culture inserts. VEGF (25 ng/ml) was placed into the lower chamber. The assembled cell culture insert chamber was then incubated at 37°C for 24 h. After removing the nonmigrating cells with a cotton swab in the upper chamber, migrated cells on the lower surface of the culture inserts were fixed with 4% paraformaldehyde, stained with hematoxylin, and photographed under a light microscope (Olympus, Tokyo, Japan). Five random fields (×200) were chosen in each insert, and the cell number was counted. Each group was tested in triplicate. All the experiments were repeated 3 times.

### Endothelial Cell Tube Formation Assay

A tube formation assay was performed as previously described (Su et al., 2010). Growth factor-reduced Matrigel (50 µl) was added to each well of chilled 96-well plates and incubated for 30 min at 37°C. Approximately 3×10^4^ cells were preincubated with various concentrations of peptide (1 nM, 10 nM, 100 nM, 1 µM, and 10 µM) at 37°C for 30 min before being seeded onto the solidified growth factor-reduced Matrigel in a 96-well plate. After incubating in media with or without 15 ng/ml of VEGF at 37°C for 6 h, tube formation was observed using an inverted phase contrast microscope (Olympus, Tokyo, Japan) and photographed with a digital camera (Olympus). Four random fields were chosen in each well, and the total tube length was quantified using the Image-Pro Plus Program (version 6.0; Media Cybernetics, Bethesda, MD). Each group was tested in triplicate. All the experiments were repeated 3 times.

### Immunofluorescence Microscopy

For immunostaining, the cells were washed with PBS and fixed with 4% paraformaldehyde on ice for 15 min. For paxillin staining, cells were permeabilized by treatment with 0.4% Triton X-100. The cells were then treated with 1% fetal bovine serum (FBS) for 1 h at room temperature and incubated with a mouse monoclonal antibody against paxillin (Cell Signaling Technology). Unbound proteins were removed by washing, and the cells were subsequently incubated with fluorescein isothiocyanate (FITC)-labeled secondary antibody for 1 h. Tetramethylrhodamine (TRITC)-conjugated phalloidin (Sigma) was used to stain the actin cytoskeleton. The coverslips were then washed three times with Tris-buffered saline (TBS) containing 0.05% Tween 20 and examined using a fluorescence microscope (Model Axiophot2, Zeiss) (Kim et al., 2004).

### Mouse OIR Assay

The mouse model of OIR was performed as previously described (Zhao et al., 2009). In our previous study (Zhao et al., 2009), we developed a small peptide KV11(YTMNPRKLFDY)from human apolipoprotein (a) KV which can effectively inhibit retinal pathologic angiogenesis at the dose of 50 mM (1 µl) in OIR model. Our newly developed peptide TKII-12 had a similar molecular weight as KV11. So we chose the dose of 50 mM (1 µl) for intravitreal injections in the OIR experiments. Neonatal C57BL/6J mice with their nursing mothers were randomly divided into five groups: room air plus PBS, room air plus TKII-12, oxygen plus PBS, oxygen plus TKII-12, and oxygen plus TKII-12S. Each group contained seven to nine pups. Briefly, on postnatal day 7 (P7), C57BL/6J mouse pups with their mothers were subjected to 75% ± 2% oxygen for 5 days. At P12, they were returned to room air and raised for another 5 days. On P12 and P14, peptides were administered by intravitreal injections at the concentration of 50 mM (1μl), respectively. Eyes of control mice were injected with 1 μl of PBS or TKII-12S (50 mM). Groups kept in room air received 1 μl of PBS or TKII-12 (50 mM) injections at the same time as those treated with oxygen. All the injections were performed in the right eyes, and the left eyes were not injected. At P17, the mice were killed, and then, their eyes were enucleated and subjected to the following examinations.

To quantitatively analyze the areas of neovascularization and the avascular zone, retinal whole mounts were stained with Alexa Fluor 568 conjugated isolectin B4 (Molecular Probes, Eugene, OR) which showed strong affinity for vascular endothelial cells and perivascular cells. Briefly, the eyes were fixed in 4% paraformaldehyde at 4°C for 8 to 12 h. Then, the neuroretina was separated from the eye cup and fixed for another 2 h. The retinas were permeabilized in 1% Triton X-100 for 30 min, and then incubated with 5 μg/ml of isolectin B4 overnight at 4°C. The retinas were rinsed three times in PBS, mounted in PBS:glycerol (2:1), and covered with a coverslip. All fluorescein images were taken by a fluorescence microscope (Carl Zeiss Meditec), with a standardized technique at a magnification of × 5. Flat-mounted retina images were assessed by image analysis software (Image-Pro Plus Program, version 6.0; Media Cybernetics, Bethesda, MD) to quantify the areas of avascular retina, retinal neovascularization, and normal vascularization. The total surface area of the retina was outlined using the outermost vessel of the arcade near the ora serrata as the border. Neovascularization, vaso-obliteration and normal vasculature were circumscribed based on their characteristic appearance, as described previously, and were normalized to the total retinal area. All images were examined in a blinded fashion.

Meanwhile, the eyes were fixed with 4% paraformaldehyde and embedded in paraffin. Ten sections, 30 μm apart, were cut sagittally through the cornea and parallel to the optic nerve and stained with hematoxylin and eosin. A blood vessel profile (BVP) was defined as an endothelial cell or a blood vessel with a lumen. BVPs were counted in the inner retina, which consists of the inner limiting membrane, the ganglion cell layer, the inner plaxiform layer, and the inner nuclear layer. Five nonoverlapping fields per section were examined by two observers in a masked fashion. The mean of all 10 counted sections yield average retinal neovascular cell nuclei per eye.

### Statistical Analysis

The experimental data were expressed as mean ± SEM from at least three independent experiments. Analysis was performed with one-way analysis of variance (ANOVA) for multiple groups. For comparison of the differences between groups, a *post hoc* least significant difference (LSD) test was used. A *p* value <0.05 was considered to be statistically significant in all cases.

## Results

### Inhibition of VEGF-Induced Endothelial Cell Proliferation by TKII-12

**Figure 2:**
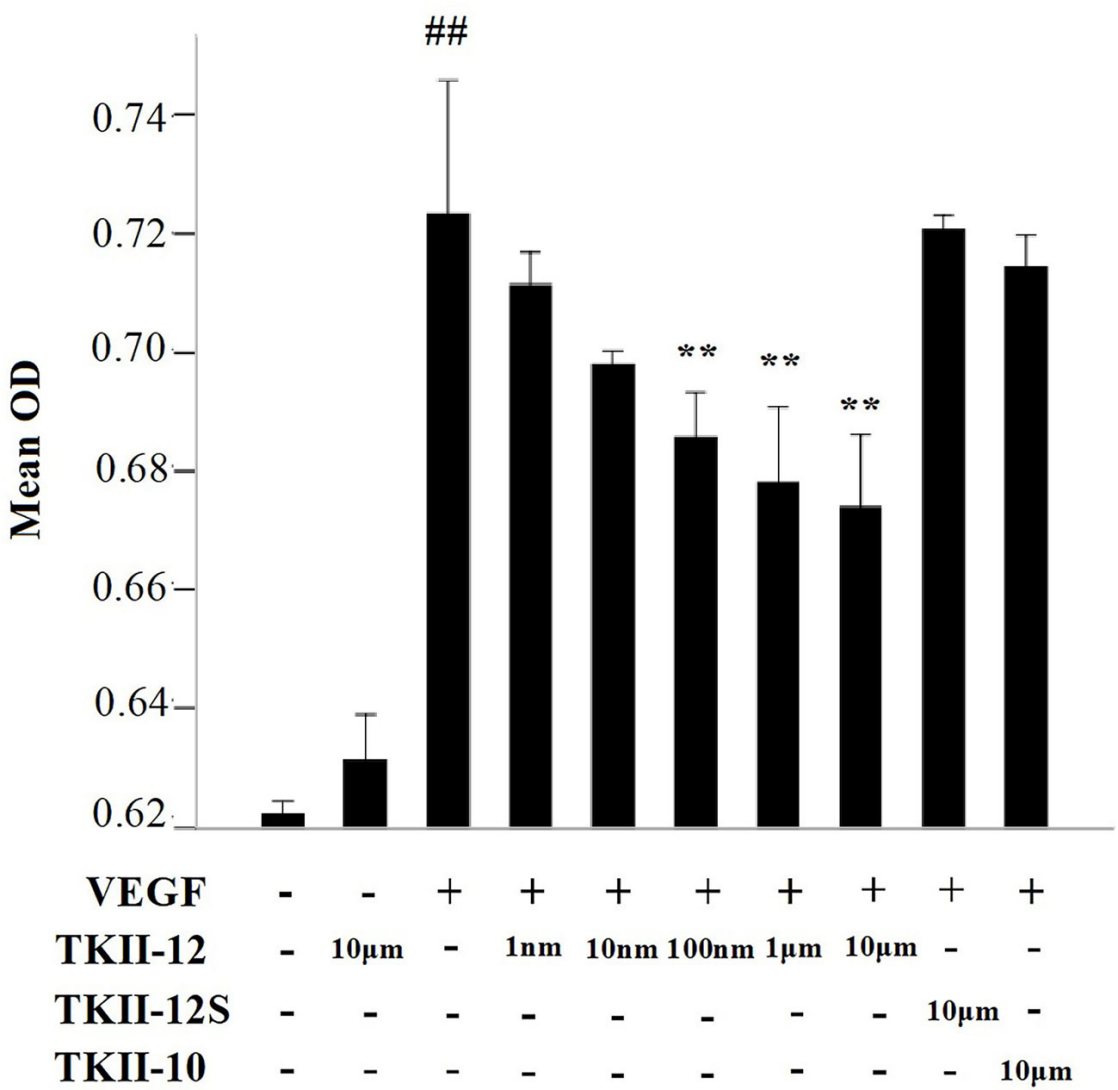
Effects of TKII-12 on vascular endothelial growth factor (VEGF)-induced (HRMECs proliferation *in vitro*. Inhibitory effect of TKII-12 on endothelial cell proliferation was assessed by MTS method 24 h after treatment. Each value represents the mean ± SEM of triplicate measurements. **p < 0.01 versus the VEGF group. ^##^*p*< 0.01 versus the control group.

In cell proliferation assay, HRMECs were exposed to increasing concentrations of the peptides for 24 h in the presence of 10 ng/ml of VEGF as mitogenic stimulus. As shown in **Figure 2**, compared with control group, TKII-12 alone without VEGF had no significant effect on HRMECs proliferation (*p* > 0.05). TKII-12 dose-dependently inhibited VEGF-induced HRMECs proliferation, and the concentration of TKII-12 required to reach 50% inhibition (ED50) was between 100 nM and 1 µM. However, TKII-10 did not show any significantly inhibitory effect on VEGF-induced HRMECs proliferation at the concentration of 10 µM, which was consistent with our previous results (Su et al., 2010). TKII-12S did not show any inhibitory effect on VEGF-induced HRMEC proliferation up to the concentration of 10 µM. Our results showed that TKII-12 can inhibit VEGF-induced HRMECs proliferation dose-dependently and sequence-dependently without significant toxic effect on endothelial cell proliferation.

### Inhibition of VEGF-Induced Endothelial Cell Migration by TKII-12

**Figure 3:**
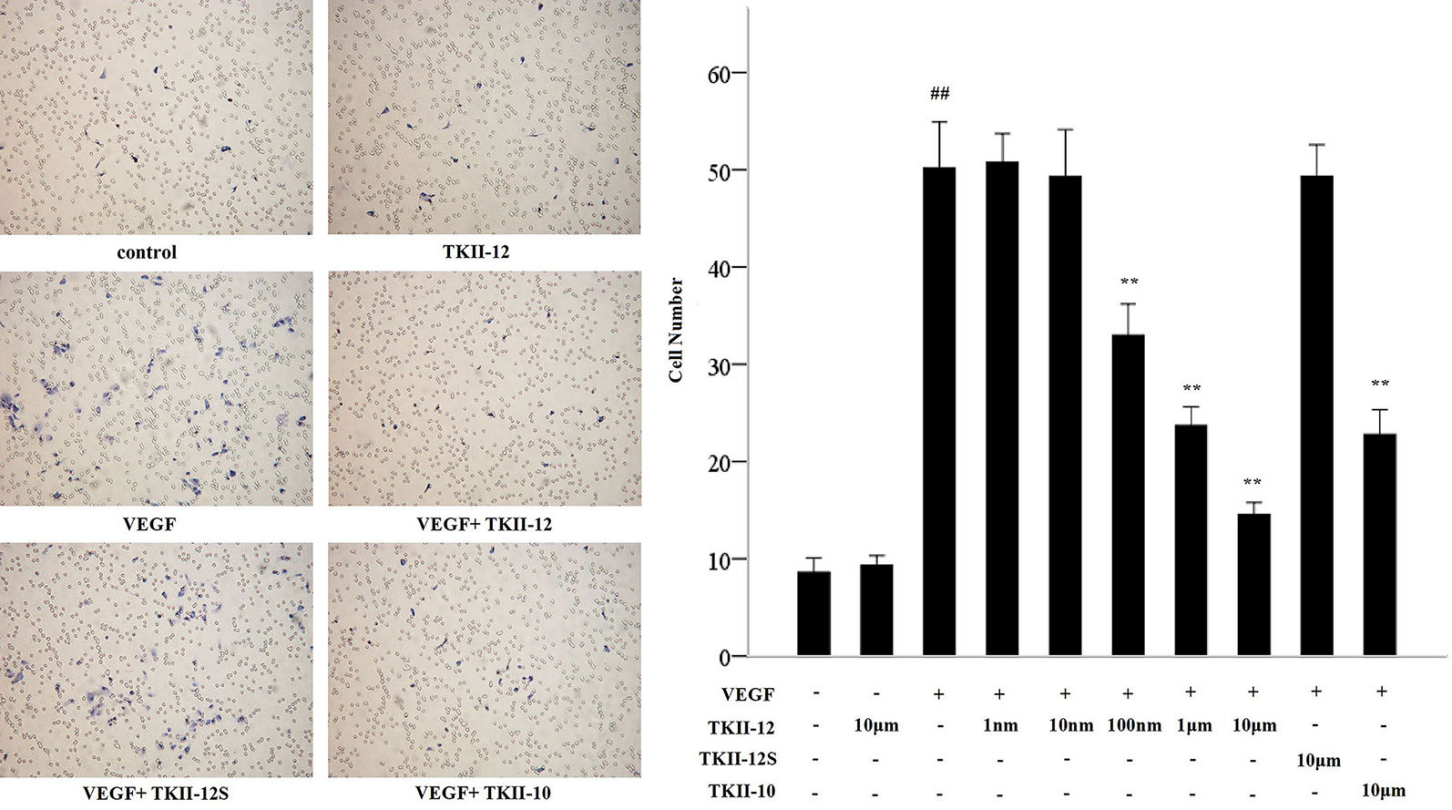
Effects of TKII-12 on vascular endothelial growth factor (VEGF)-induced HRMECs migration *in vitro*. Inhibitory effect of TKII-12 on endothelial cell migration toward VEGF was assessed by a Transwell chamber 24 h after treatment. **p < 0.01 versus the VEGF group. ^##^*p* < 0.01 versus the control group.

Compared with control group, TKII-12 alone without VEGF had no significant effect on HRMECs migration (*p* > 0.05). Compared with the control group, the number of migration cells in the VEGF group increased significantly (*p* < 0.05). As shown in **Figure 3**, TKII-12 potently inhibited VEGF-induced HRMECs migration in a dose-dependent as well as sequence-dependent manner with an estimated ED50 value between 100 nM and 1 µM, whereas TKII-12S had no such activity. TKII-10 inhibited VEGF-induced HRMECs migration at the concentration of 10 µM, but the inhibitory effect less effective than TKII-12.

### Inhibition of VEGF-Induced Endothelial Cell Tube Formation by TKII-12

**Figure 4:**
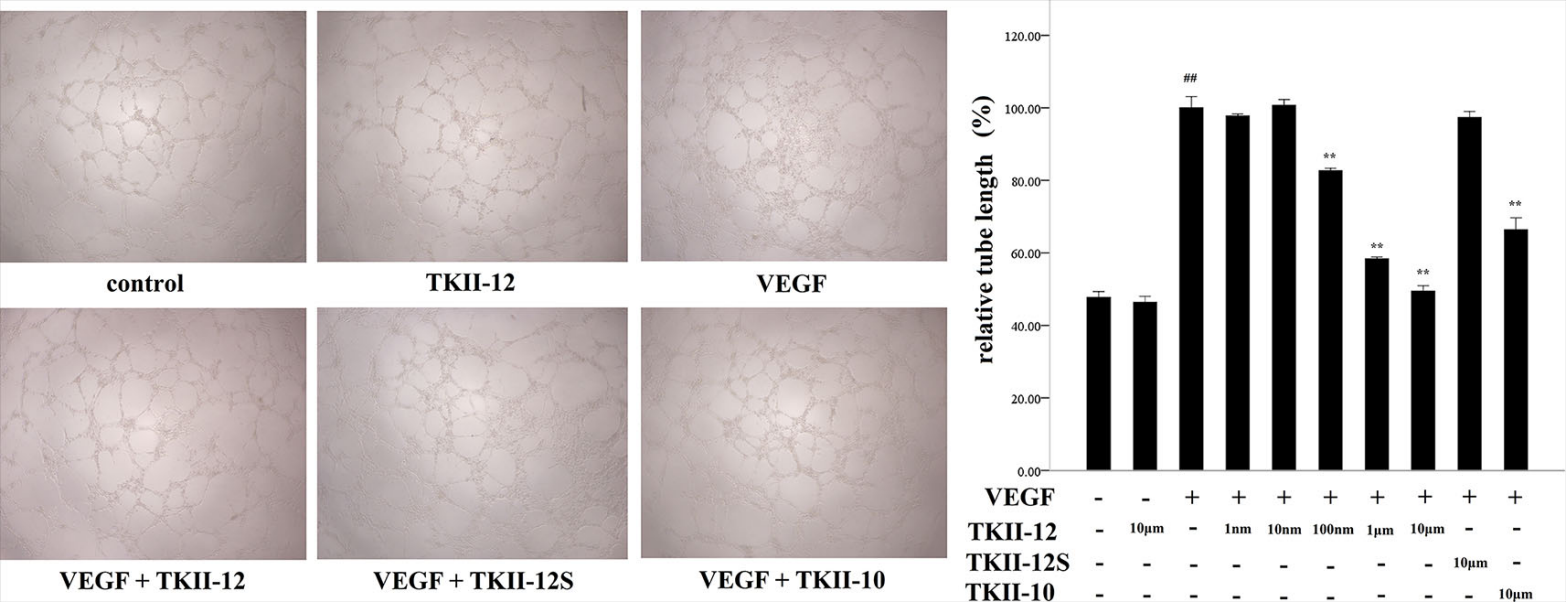
Effects of TKII-12 on vascular endothelial growth factor (VEGF)-induced HRMECs tube formation on Matrigel. The left panel is the representative phase-contrast micrographs of HRMECs exposed to different culture medium at 6 h (magnification 20×). The right panel presents the quantitative graph of the relative percentage of tube length. Each value represents the mean ± SEM of triplicate measurements. **p < 0.01 versus the VEGF group. ^##^*p* < 0.01 versus the control group.

As shown in **Figure 4**, compared with control group, TKII-12 alone without VEGF had no significant effect on HRMECs tube formation (*p* > 0.05). VEGF (15 ng/ml) strongly stimulated HRMECs cell tube formation on Matrigel, compared with control group (*p* < 0.05). TKII-12 inhibited VEGF-induced HRMECs tube formation on Matrigel dose dependently, and at 10 µM concentration TKII-12 almost completely inhibited VEGF-induced HRMECs cells tube formation on Matrigel. TKII-12 inhibited VEGF-induced HRMECs tube formation sequence-dependently as TKII-12S was inactive. TKII-10 also inhibited VEGF-stimulated HRMECs tube formation, but the inhibitory effect was not as strong as TKII-12 at the concentration of 10 µM.

### Inhibition of Actin Stress Fibers and Focal Adhesions Formation by TKII-12

**Figure 5:**
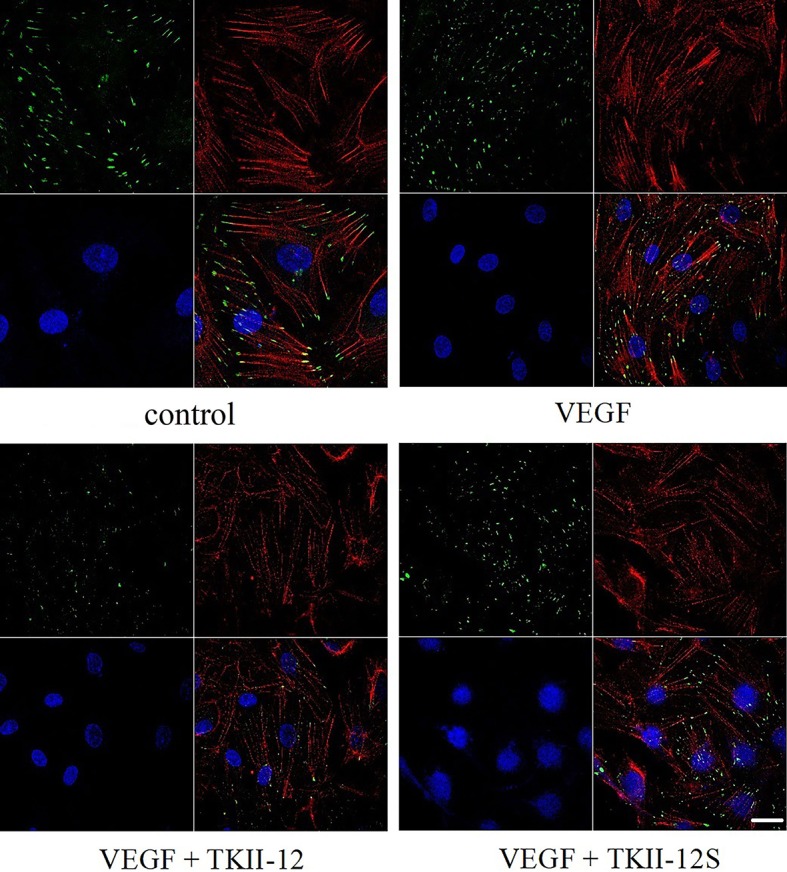
Effects of TKII-12 on vascular endothelial growth factor (VEGF)-induced formation of actin stress fibers and focal adhesion complexes in HRMECs. HRMECs were immunostained with an antivinculin antibody and stained with TRITC-conjugated phalloidin and DAPI. The scale bar is 20 μm.

The dynamic formation of actin stress fibers and focal adhesions played a crucial role in the regulation of cell adhesion and movement. As shown in **Figure 5**, VEGF significantly increased the formation of actin stress fibers and focal adhesions in vascular endothelial cells. Consistent with the effects of TKII-12 on VEGF-induced cell migration and tube formation, the formation of actin stress fibers and focal adhesions induced by VEGF was greatly decreased by TKII-12. The inhibitory effect was sequence-dependently as TKII-12S was inactive.

### Inhibition of Oxygen-Induced Neovascular Retinopathy by TKII-12

**Figure 6:**
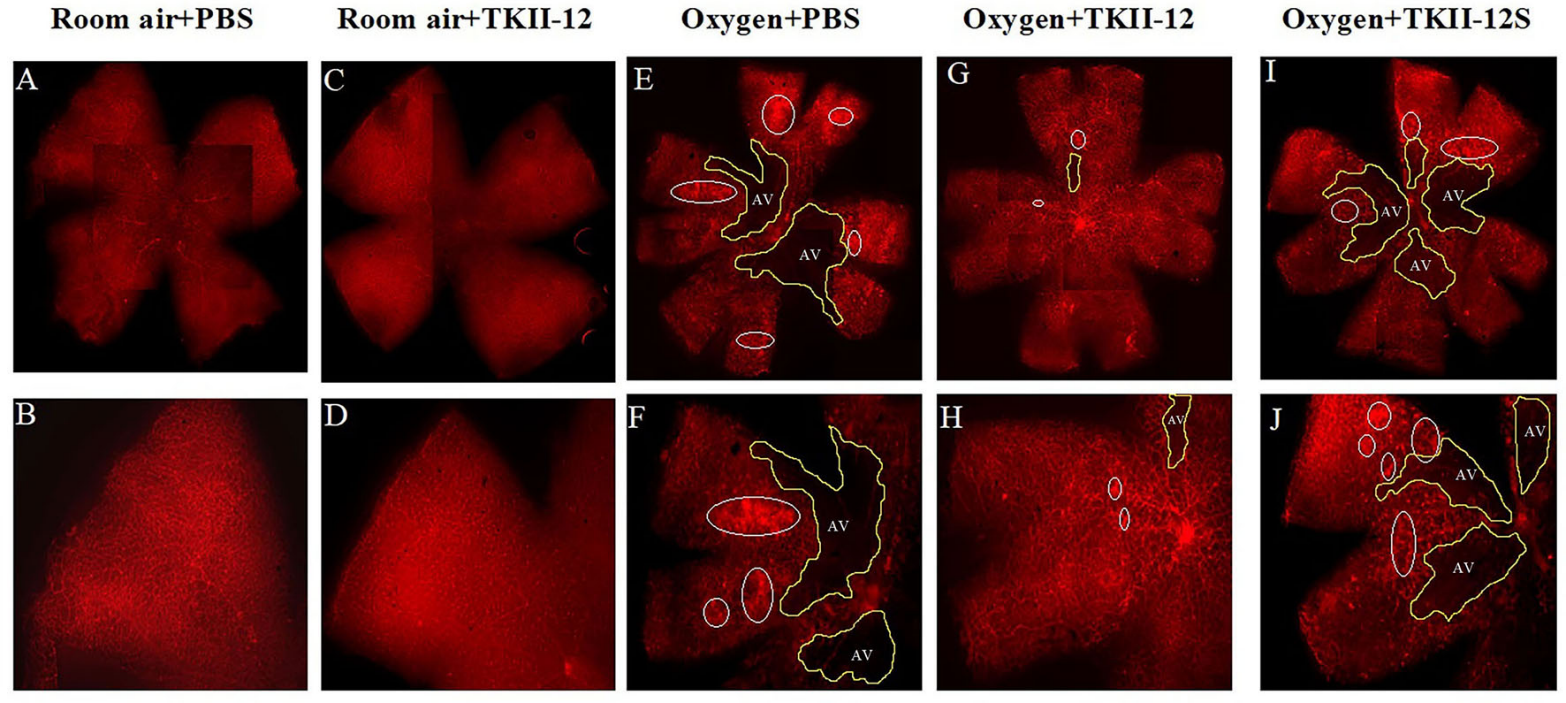
Retina flat-mounts stained with isolectin B4 from each group at P17. The retinal vasculature in the room air plus TKII-12 group **(C, D)** was no different from that of the room air plus PBS group **(A, B)**. Oxygen plus phosphate-buffered saline (PBS) **(E, F)** and oxygen plus the scrambled peptide **(G, H)** groups exhibited a remarkable avascular region and pathologic neovascular tufts. Oxygen plus TKII-12 group **(I, J)** showed a significant reduction in neovasculature and avascular region. Top row: whole retina montages created by four overlapping images containing one quadrant each; bottom row: a single image of the whole flat-mount, showing one quadrant. White circle: neovascular tufts. Yellow area: avascular region. Magnification, ×5.

**Figure 7:**
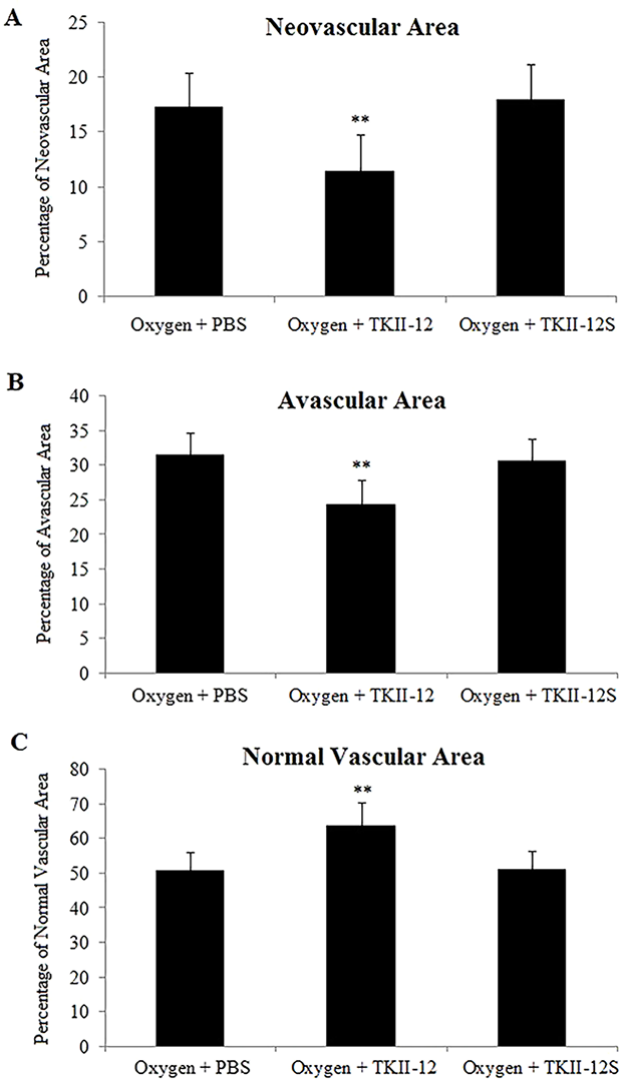
Quantitative analysis of the effects of TKII-12 on oxygen-induced retinopathy in a mouse model. Quantification of neovascularization **(A)**, avascularization **(B)**, and normal vascularization **(C)** of each group were performed at P17. **p < 0.01 versus the oxygen plus phosphate-buffered saline (PBS) group.

The inhibitory effect of oxygen-induced retinal neovascularization by TKII-12 was quantitatively analyzed by isolectin B4 staining. The isolectin-stained retinal whole mounts were used to evaluate the neovascular, avascular, and normal vascular areas at P17. As shown in **Figure 6**, retinas of the room air plus TKII-12 group demonstrated normal retinal vascular development, without obvious differences from that of the room air plus PBS group, indicating that TKII-12 did not inhibit retinal vascular development under normal condition. Retinas of the oxygen plus PBS group exhibited obvious central vascular obliteration and extensive preretinal neovascular tuft formation located mainly at the junction between the central avascular and peripheral vascularized regions. Intravitreous TKII-12 injection significantly improved the pathological vascular responses. Compared with the oxygen plus PBS group, TKII-12 dramatically reduced the pericentral neovascular tuft area by up to 34% (from 17.37% ± 3.07% to 11.45% ± 3.37% of the whole retinal area) (*p* < 0.01). Meanwhile, TKII-12 also significantly reduced the central avascular area by up to 23% (from 31.70% ± 2.98% to 24.51% ± 3.36% of the whole retinal area) (*p* < 0.01), with a significant increase in the percentage of peripheral normal vascular area (from 50.93% ± 5.12% to 64.04% ± 6.55% of the whole retinal area) (*p* < 0.01) (**Figure 7**). These data indicated that TKII-12 potently inhibited pathological retinal neovascularization and enhanced physiological vascular regrowth in OIR. In contrast, intravitreous TKII-12S injection demonstrated no significant improvement in preretinal neovascular tuft formation and central vascular obliteration (*p* > 0.05), indicating that the angiogenic inhibitory effect of TKII-12 was sequence-dependent.

**Figure 8:**
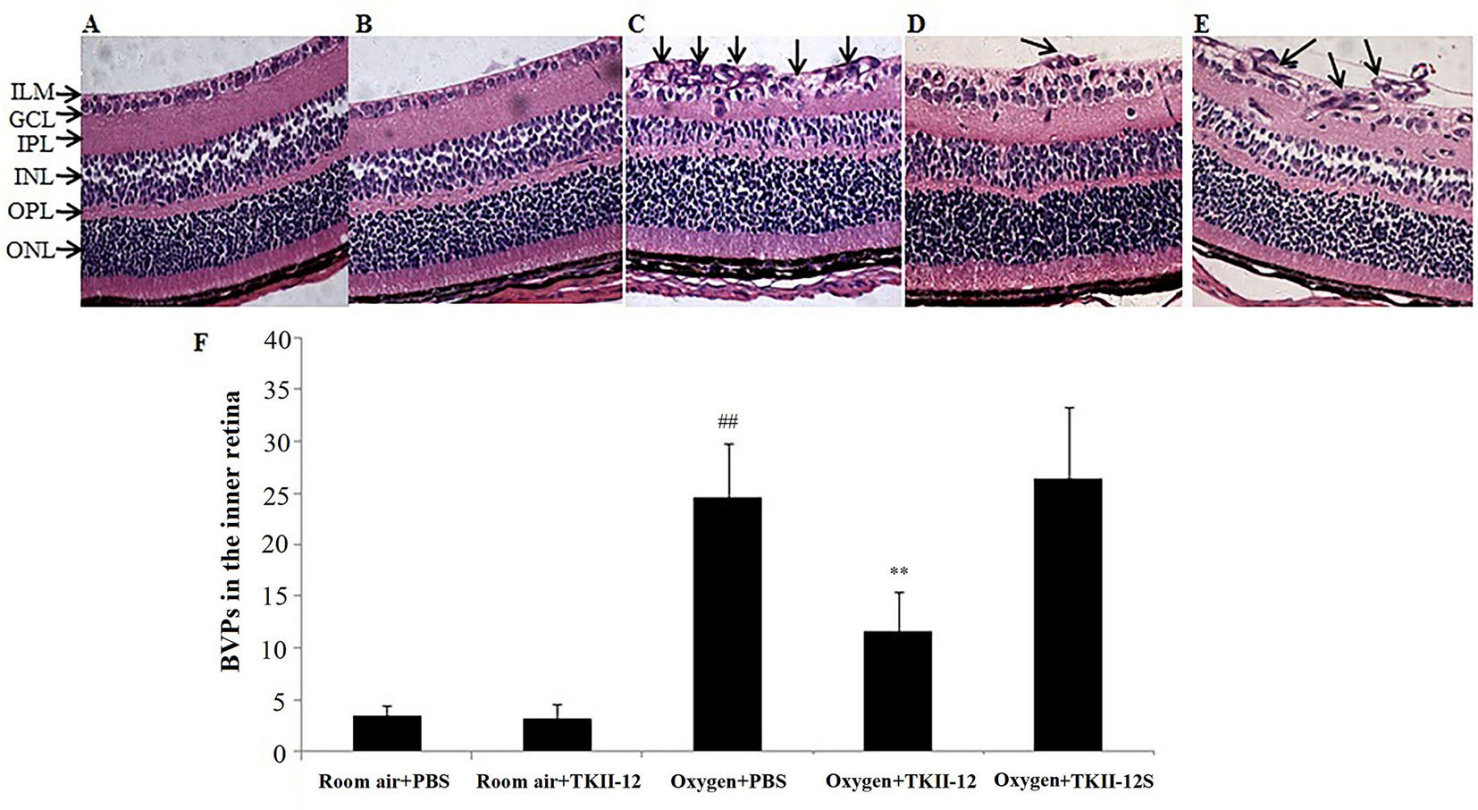
Inhibitory effect of TKII-12 on oxygen-induced retinopathy in mice. Paraffin-embedded sections of the retina stained with hematoxylin and eosin. **(A)** Room air plus phosphate-buffered saline (PBS); **(B)** room air plus TKII-12; **(C)** oxygen plus PBS; **(D)** oxygen plus TKII-12; **(E)** oxygen plus the scrambled peptide. Arrows: blood vessel profiles (BVPs) in the inner retina or extending from the retina into the vitreous cavity. Magnification, ×100. **(F)** Quantification of BVPs in the inner retina. ^##^*p* < 0.01 versus the room air plus PBS group. **p < 0.01 versus the oxygen plus PBS group.

Histological examinations of the retinal sections further confirmed the preretinal neovascular tuft formation in the retinal flat mounts (**Figure 8**). The degree of retinal neovascular formation was quantified by counting BVPs in the inner retina in at least 10 sections per eye. Retinas in the room air plus TKII-12 group demonstrated few preretinal neovascular tuft, with no significant difference in BVP number compared with the room air plus PBS group (*p* > 0.05). Retinas in the oxygen plus PBS group showed obvious vascular lumen formation in the inner retina, and the BVP number significantly increased compared with the room air plus PBS group (*p* < 0.01). The BVP number significantly decreased in the oxygen plus TKII-12 group compared with the oxygen plus PBS group (*p* < 0.01). In contrast, the BVP number in the oxygen plus TKII-12S group showed no significant difference with that in the oxygen plus PBS group (*p* > 0.05), indicating that TKII-12 inhibited oxygen-induced retinal neovascularization sequence-dependently.

## Discussion

Retinal neovascularization is a major cause of vision loss in humans, and the potential therapeutic effects of angiogenic inhibitors in neovascular retinopathy are under intensive investigation. In this study, we observed that a small peptide, TKII-12, derived from human tissue-type plasminogen kringle 2 effectively inhibited VEGF-induced HRMECs proliferation, migration, and tube formation dose-dependently as well as sequence-dependently *in vitro*. TKII-12 decreased the formation of actin stress fibers and focal adhesions in vascular endothelial cells. Furthermore, TKII-12 effectively inhibited retinal neovascularization in a mouse OIR model. TKII-12 is a promising angiogenic inhibitor for the treatment of neovascular retinopathy.

In an effort to develop angiogenic inhibitors, we divided human t-PA kringle 2 into 4 nonoverlapping peptides (shown in **Figure 1**). In a series of studies, we analyzed the antiangiogenic effects of all these four peptides. Our previous study demonstrated that TKII-10 (Arg54-Trp63) inhibited VEGF stimulated migration and tube formation of vascular endothelial cells *in vitro* and corneal neovascularization *in vivo* (Su et al., 2010). In this study, we demonstrated that TKII-12 (His65-Tyr76) inhibited VEGF-induced HRMECs proliferation, migration, and tube formation *in vitro* and pathological retinal neovascularization *in vivo*. Compared with TKII-10, the antiangiogenic effect of TKII-12 was stronger and more comprehensive. Besides TKII-12 and TKII-10, peptide Tyr2-Ser21 from human t-PA kringle 2 did not show any antiangiogenic effect *in vitro*. Peptide Leu23-Tyr52 from human t-PA kringle 2 inhibited VEGF-induced endothelial cell migration and tube formation *in vitro*, but the antiangiogenic effect was not as strong as TKII-12. Moreover, the solubility of peptide Leu23-Tyr52 was not as good as the other three peptides. Thus, we fully investigated the most promising antiangiogenic peptide TKII-12.

Angiogenesis is a multistep process that includes vascular endothelial cell proliferation and migration, capillary tube formation, and extracellular matrix degeneration and remodeling. Alteration of the cytoskeleton and the temporal-spatial organization of cell adhesion structures are central events in endothelial cell migration (Shen and Guan, 2001; Wozniak et al., 2004). Our results indicated that TKII-12 suppressed VEGF-stimulated formation of focal adhesion complexes and the consequent organization of actin stress fibers. The recombinant protein TK1-2 (kringle 1-2 from human t-PA) inhibited angiogenesis partly by the interaction with integrin α2β1 (Kim et al., 2003; Kim et al., 2008). TKII-12 was derived from the extended antiparallel β-sheet within the t-PA kringle 2 domain which also demonstrated antiangiogenic activity (Byeon et al., 1991; Carroll et al., 2005). Another peptide, TP-7, derived from t-PA kringle 2 inhibited VEGF or FGF-induced phosphorylation of ERK1/2 and FAK (Kim et al., 2017). Future studies are needed to identify the exact antiangiogenic mechanism of TKII-12.

Toxicity investigation is a critical part of antiangiogenic drug development. In this study, TKII-12 alone without VEGF did not show any potential toxicity to vascular endothelial cells as it had no significant effect on cell proliferation, migration or tube formation *in vitro*. In the OIR study, intravitreal TKII-12 injection in the room air plus TKII-12 group showed no obvious qualitative abnormalities such as vessel dilation, tortuosity, leakage, or hemorrhages, indicating that TKII-12 did not affect the normal retinal vessel development. These results indicated that TKII-12 had no obvious toxicity to the retinal vessel development, and its inhibitory effect on angiogenesis was specific to pathological retinal neovascularization. Our previous report also proved the safety of small peptides to retinal tissues. Intravitreal administration of KV11, even up to the concentration of 200 mM, did not display notable retinal toxicity as evaluated by electrophysiological and ultrastructural examinations (Zhao et al., 2009). Small peptides also show sufficient penetration capabilities. FITC-labeled KV11 could rapidly penetrate the whole retina. However, even after high-dose intravitreal injection, the plasma concentration of KV11 was still too low to induce evident systemic adverse reactions. TKII-12 has a similar molecular weight, isoelectric point, net charge, and good water solubility as KV11. Therefore, TKII-12 could be expected to have a similar favorable safety profile and penetration capability as KV11.

In previous studies, Kim et al. reported the recombinant protein TK1-2 and the t-PA kringle 2 domain alone inhibited angiogenesis *in vitro* and *in vivo* (Kim et al.,2003; Carroll et al., 2005; Kang et al., 2006; Kim et al., 2008). Kim also reported a peptide derived from t-PA kringle 2 (named TP-7) that inhibited angiogenesis and corneal neovascularization (Kim et al., 2017). TP-7 was derived from an extended antiparallel β-sheet motif of t-PA kringle 2. TP-7 can in part affect the integrin α2β1-dependent pathway and target VEGF and non-VEGF pathways. Our designed peptides (TKII-10 and TK11-12) were also derived from t-PA kringle 2, but they were designed using the cysteine in disulfide bonds for cleavage sites. TKII-10 can inhibit VEGF-stimulated migration and tube formation of HUVECs *in vitro* and effectively inhibited angiogenesis in chick chorioallantoic membrane and VEGF-induced corneal neovascularization. TKII-12 could effectively inhibit retinal angiogenesis *in vitro* and *in vivo* by eliminating the formation of focal adhesion complexes and the organization of actin stress fibers. Both of these peptides may serve as prototypes for antiangiogenic drug development.

While the widely-used anti-VEGF therapy has revolutionized the treatment of retinal neovascular diseases, it is still with some limitations. Anti-VEGF therapy effectively decreased retinal and choroidal neovascular lesions; however, it can lead to ocular fundus fibrotic scarring and largely hinder the ultimate vision acuity improvement (Gemenetzi et al, 2017). With long-term studies, anti-VEGF resistance is now coming to our mind (Yang et al, 2016). This highlights the need for the development of new antiangiogenic drugs which can target different mechanism and reduce the necessity of invasive and repetitive intravitreous injections. Compared to proteins, low-molecular-weight small peptides, such as TKII-12, possessed its own incomparable superiority with easier and inexpensive synthesizing methods, improved consistency between batches, lower immunogenicity, higher solubility in water, and better penetrating abilities. Considering the hurdles in the clinical applications of large proteins, small peptides may provide promising candidates for new ocular antiangiogenic therapy.

## Conclusion

In summary, TKII-12 can effectively inhibit VEGF-induced human retinal microvascular endothelial cell proliferation, migration, and tube formation *in vitro* and eliminate oxygen-induced retinal neovascularization *in vivo* by decreasing the formation of actin stress fibers and focal adhesions. TKII-12 may provide an effective approach for pathological retinal neovascularization therapy.

## Data Availability Statement

The raw data supporting the conclusions of this article will be made available by the authors, without undue reservation, to any qualified researcher.

## Ethics Statement

The animal study was reviewed and approved by Shanghai General Hospital.

## Author Contributions

LS, QS, and XX designed the experiment. LS, YS, and QS performed the experiment. LS analyzed data and wrote the manuscript. XX conceived and supervised the study.

## Funding

This project was sponsored by the National Key R&D Program of China (No. 2016YFC0904800) and the Program (No. 81302683 and 81570851) of the National Natural Science Foundation of China.

## Conflict of Interest

The authors declare that the research was conducted in the absence of any commercial or financial relationships that could be construed as a potential conflict of interest.
